# MEGA-RAG: a retrieval-augmented generation framework with multi-evidence guided answer refinement for mitigating hallucinations of LLMs in public health

**DOI:** 10.3389/fpubh.2025.1635381

**Published:** 2025-10-08

**Authors:** Shan Xu, Zhaokun Yan, Chengxiao Dai, Fan Wu

**Affiliations:** ^1^Shanghai Institute of Infectious Disease and Biosecurity, School of Public Health, Fudan University, Shanghai, China; ^2^China Academy of Information and Communications Technology, Beijing, China; ^3^Faculty of Engineering, University of Sydney, Sydney, NSW, Australia

**Keywords:** public health, AI in healthcare, LLM hallucinations, medical question answering, RAG

## Abstract

**Introduction:**

The increasing adoption of large language models (LLMs) in public health has raised significant concerns about hallucinations-factually inaccurate or misleading outputs that can compromise clinical communication and policy decisions.

**Methods:**

We propose a retrieval-augmented generation framework with multi-evidence guided answer refinement (MEGA-RAG), specifically designed to mitigate hallucinations in public health applications. The framework integrates multi-source evidence retrieval (dense retrieval via FAISS, keyword-based retrieval via BM25, and biomedical knowledge graphs), employs a cross-encoder reranker to ensure semantic relevance, and incorporates a discrepancy-aware refinement module to further enhance factual accuracy.

**Results:**

Experimental evaluation demonstrates that MEGA-RAG outperforms four baseline models [PubMedBERT, PubMedGPT, standalone LLM, and LLM with standard retrieval-augmented generation (RAG)], achieving a reduction in hallucination rates by over 40%. It also achieves the highest accuracy (0.7913), precision (0.7541), recall (0.8304), and F1 score (0.7904).

**Discussion:**

These findings confirm that MEGA-RAG is highly effective in generating factually reliable and medically accurate responses, thereby enhancing the credibility of AI-generated health information for applications in health education, clinical communication, and evidence-based policy development.

## 1 Introduction

AI has seen widespread adoption in public health, applied to tasks such as disease prediction, clinical decision-making, outbreak surveillance, and large-scale infoveillance, leveraging diverse data sources including electronic health records, epidemiological reports, and social media streams (1–7). Public health itself encompasses a wide range of activities, from disease management and improving health outcomes to emergency response (8–10).

Despite the promise of AI, LLM in public health face significant challenges due to hallucinations—plausible yet factually incorrect outputs that can mislead clinical guidance and policy (11–15). While existing strategies such as domain-specific fine-tuning, adversarial training, and retrieval-augmented generation with structured knowledge bases provide some mitigation, they offer only partial solutions (16–18). Recent research has explored one-shot hallucination detection using hidden states, attention maps, and output probabilities (19, 20). Knowledge distillation with soft labels during supervised fine-tuning has also been shown to improve factual grounding while maintaining general NLP performance (21–23). However, comprehensive methods specifically targeting hallucination mitigation in public health are still lacking, which motivated the development of MEGA-RAG (24, 25).

## 2 Related work

Significant prior work has integrated knowledge graphs into the RAG framework, particularly in the field of biomedicine. For instance, KG-RAG introduces a token-optimized algorithm for embedding biomedical KGs into the RAG framework, improving the quality of retrieved information (26). Similarly, KRAGEN enhances RAG by incorporating a knowledge graph framework specifically tailored for biomedical problem-solving, further advancing its applicability in clinical contexts (27). Other approaches, such as Zebra-Llama, combine context-aware large language models with external knowledge bases to democratize knowledge related to rare diseases, improving accessibility and understanding (28). Additionally, MedRAG uses knowledge graph-elicited reasoning to refine retrieval-augmented generation, enhancing the accuracy and reliability of healthcare applications (29). These contributions represent significant strides in leveraging knowledge graphs to tackle challenges like hallucinations, while also strengthening the robustness of clinical decision-making systems in public health.

## 3 Methods

### 3.1 MEGA-RAG framework

**Figure 1:**
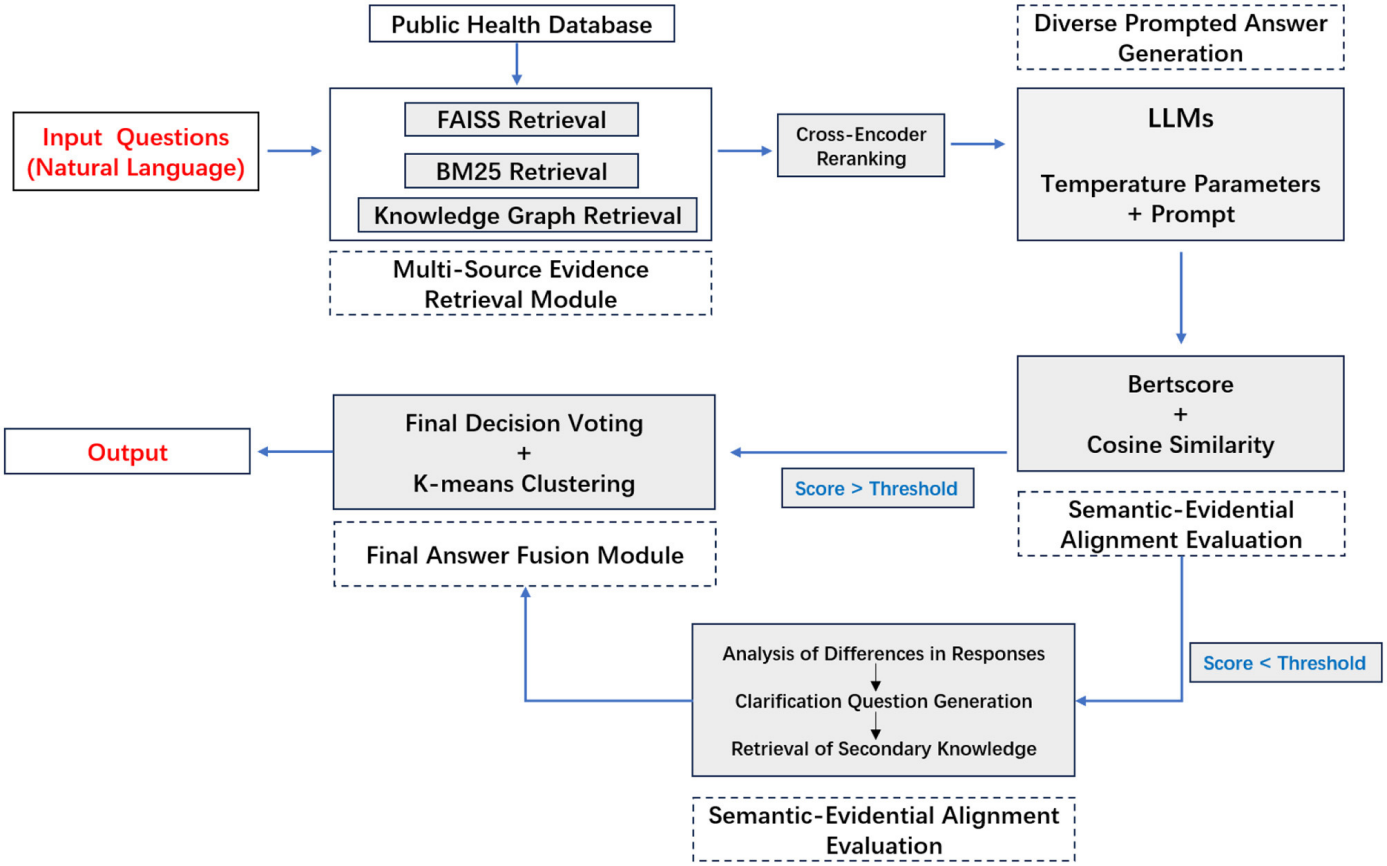
MEGA-RAG framework diagram.

**Figure 2:**
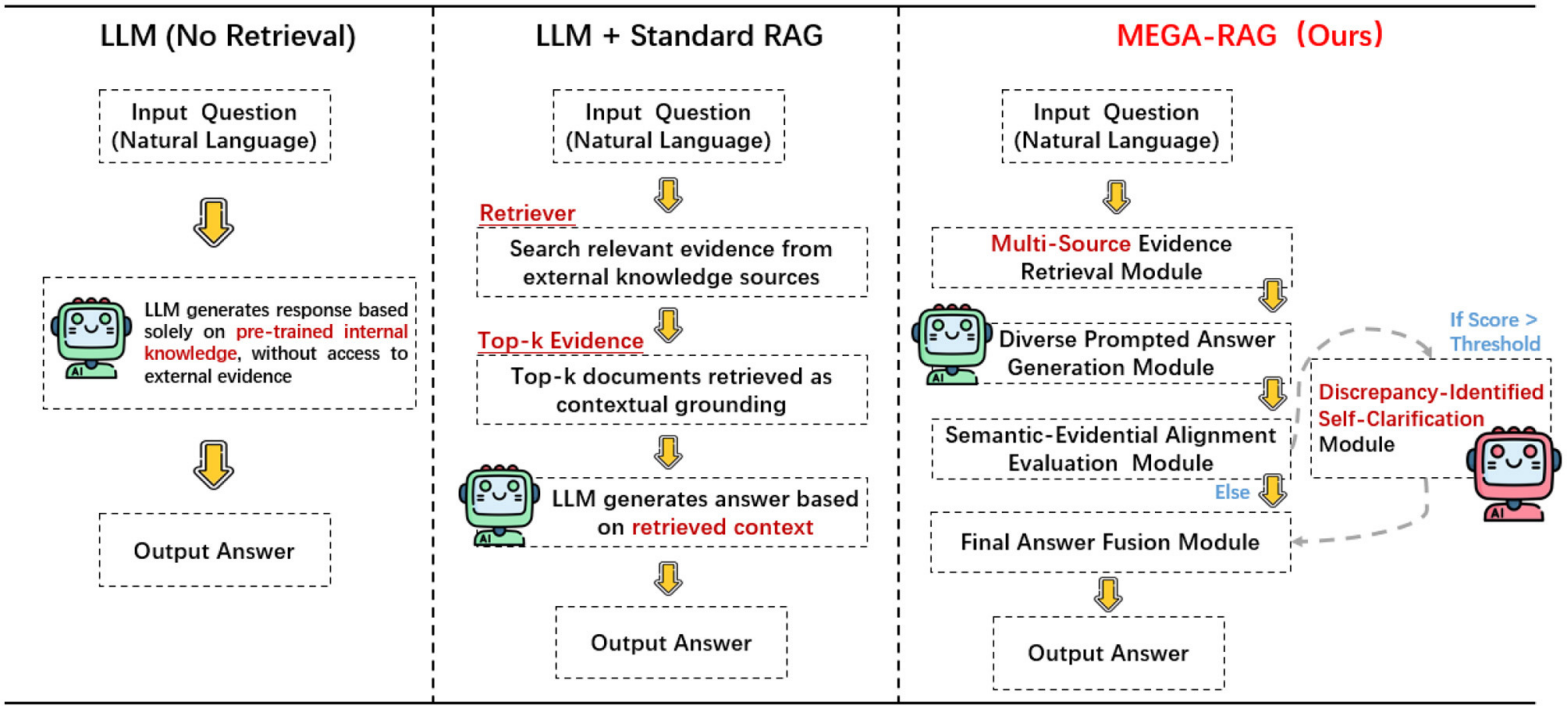
Mechanism comparison of LLM, LLM+RAG, and MEGA-RAG.

We propose **MEGA-RAG**, a novel retrieval-augmented question answering (QA) framework specifically tailored for the biomedical and public health domains, where **accuracy**, **interpretability**, and **evidence grounding** are critical. As illustrated in Figures 1, 2, conventional large language models (LLMs) generate answers based solely on their internal knowledge, which increases the risk of hallucinations and factual inconsistencies. Traditional retrieval-augmented generation (RAG) frameworks mitigate this issue by retrieving supporting documents; however, they typically rely on a single retrieval pass and lack mechanisms for evaluating answer consistency or handling conflicting evidence. In contrast, **MEGA-RAG** integrates a four-stage architecture designed to overcome these limitations.

First, the **Multi-Source Evidence Retrieval (MSER) Module** aggregates heterogeneous information through **FAISS-based dense retrieval**, **BM25 keyword search**, and **biomedical knowledge graphs**, thereby improving both recall and factual grounding (30–32). Second, the **Diverse Prompted Answer Generation (DPAG) Module** generates multiple candidate answers via prompt-based LLM sampling, which are then reranked using **cross-encoder relevance scoring** (33). Third, the **Semantic-Evidential Alignment Evaluation (SEAE) Module** evaluates answer consistency by calculating cosine similarity and BERTScore-based alignment with the retrieved evidence (34–36). Finally, the **Discrepancy-Identified Self-Clarification (DISC) Module** detects semantic divergence across answers, formulates clarification questions, and performs secondary retrieval with knowledge-guided editing to resolve conflicts.

This multi-evidence, multi-stage refinement process enables **MEGA-RAG** to deliver more accurate, trustworthy, and policy-aligned responses compared to both standard LLMs and conventional RAG methods.

### 3.2 Multi-source evidence retrieval module

To enhance factual accuracy and evidence coverage in public health question answering, **MEGA-RAG** retrieves data concurrently from three complementary, authoritative sources:

- **PubMed articles and abstracts:** Retrieves peer-reviewed biomedical literature, including randomized controlled trials, systematic reviews, and clinical practice guidelines. Structured abstracts and full-text articles are harvested to ensure that answers are based on rigorously vetted research.
- **WHO IRIS database:** Leverages the World Health Organization's Institutional Repository for Information Sharing (IRIS), which houses official public health policies, technical guidance, surveillance reports, and emergency response frameworks. IRIS provides up-to-date, globally harmonized documents on disease prevention, outbreak management, and health system strengthening.
- **CPubMed-KG knowledge graph:** Utilizes a curated graph of biomedical triples (head, relation, tail) that encodes mechanistic links between pathogens, interventions, host responses, and epidemiological outcomes. This structured layer supports causal reasoning and semantic disambiguation, ensuring that complex relationships—such as transmission pathways or drug-disease interactions—are explicitly represented.

#### 3.2.1 Dense semantic retrieval (FAISS)

The dense semantic retrieval module employs FAISS to perform approximate nearest-neighbor search over a large embedding index. By encoding both queries and documents into high-dimensional vector representations, typically using a pre-trained language model, FAISS can quickly identify semantically similar items even when they share few or no exact keywords. This approach captures conceptual similarity, enabling the system to retrieve contextually relevant information based on meaning rather than lexical overlap.

Mathematically, given a query vector **q** and a set of document vectors **d**_*i*_, FAISS performs the following nearest-neighbor search:


(1) $$\begin{array}{l} {\text{argmin}}_{i}||\text{q}-{\text{d}}_{i}|{|}_{2} \end{array}$$

where ||·||_2_ represents the Euclidean distance between the query vector and the document vector, and *i* represents the index of the closest document.

#### 3.2.2 Sparse lexical retrieval (BM25)

The sparse lexical retrieval component uses the BM25 algorithm to rank documents according to term frequency and inverse document frequency. BM25 excels at finding exact or partial keyword matches, making it effective for queries where precise terminology matters. By combining document length normalization and term-weighting heuristics, BM25 ensures that highly relevant documents containing the query terms in important contexts are prioritized.

The BM25 ranking function is given by:


(2) $$\begin{array}{l} \text{score}\left(d,q\right)=\underset{t\in q}{\sum }\text{IDF}\left(t\right)\frac{f\left(t,d\right)\left(k_{1}+1\right)}{f\left(t,d\right)+k_{1}\left(1-b+b\frac{\left|d\right|}{avgdl}\right)} \end{array}$$

where *f*(*t, d*) is the term frequency of term *t* in document *d*, |*d*| is the length of the document, *avgdl* is the average document length, *k*_1_ and *b* are free parameters, and IDF(*t*) is the inverse document frequency of term *t*.

#### 3.2.3 Knowledge graph triple retrieval

The knowledge graph retrieval component accesses structured biomedical facts represented as triples (*e*_1_, *r, e*_2_), where *e*_1_ and *e*_2_ denote entities and *r* denotes the relation connecting them. Unlike text-based retrieval, which relies on lexical or semantic similarity, this module enables explicit reasoning over mechanistic associations such as pathogen–host interactions or drug–disease relationships.

Given a natural language query *q*, the system first encodes *q* using a pre-trained language model. A lightweight mapping layer then aligns query tokens to the ontology of the knowledge graph, ensuring that linguistic expressions in *q* correspond to valid schema elements (entities or relations) in *G*. This mapping process translates unstructured natural language into graph-compatible representations, enabling downstream structured queries.

To improve entity alignment, we incorporate **named entity recognition (NER)** and **entity linking**. NER identifies mentions of biomedical entities in *q*, while entity linking resolves these mentions to canonical nodes in the knowledge graph by leveraging contextual embeddings, surface-form similarity, and frequency priors. This step is essential for disambiguating terms that may refer to multiple concepts (e.g., “SARS” as a syndrome vs. the coronavirus pathogen), thereby ensuring that retrieved triples are both semantically and clinically accurate.

Formally, the retrieval process extracts triples that satisfy both semantic and entity alignment constraints:


(3) $$\begin{array}{l} T_{q}=\left\{\left(e_{1},r,e_{2}\right)\in G:\text{match}\left(q,e_{1},e_{2},r\right)\right\} \end{array}$$

where the match function can be decomposed as:


(4) $$\begin{array}{l} \text{match}\left(q,e_{1},e_{2},r\right)=f_{\text{sem}}\left(q,r\right)\cdot f_{\text{align}}\left(q,e_{1},e_{2}\right), \end{array}$$

with *f*_sem_(*q, r*) measuring the semantic compatibility between the query and relation *r*, and *f*_align_(*q, e*_1_, *e*_2_) verifying whether the entities (*e*_1_, *e*_2_) are consistent with NER and entity linking results.

By integrating schema mapping, semantic matching, and entity alignment, the knowledge graph retrieval module provides logically structured, unambiguous, and causally meaningful evidence that complements dense and sparse retrieval. This ensures that MEGA-RAG can not only capture surface-level similarity but also reason over biomedical mechanisms explicitly encoded in the graph.

#### 3.2.4 Evidence merging and re-ranking

After retrieving candidate passages and triples from the dense, sparse, and graph-based modules, the evidence merging component consolidates all results into a unified set. A re-ranking step then orders these items according to a composite relevance score that may include semantic similarity, lexical match quality, source authority, and recency. This ensures that the final evidence context is both coherent and highly relevant to the original query before being used for downstream tasks.

Let *S*_dense_, *S*_lexical_, and *S*_graph_ be the sets of retrieved results from the dense, lexical, and graph-based modules, respectively. The unified set *S* is formed as:


(5) $$\begin{array}{l} S=S_{\text{dense}}\cup S_{\text{lexical}}\cup S_{\text{graph}} \end{array}$$

Each item in *S* is then assigned a composite relevance score *R*_*i*_ that combines various factors:


(6) $$\begin{array}{l} R_{i}=\alpha \cdot S_{\text{dense}}\left(i\right)+\beta \cdot S_{\text{lexical}}\left(i\right)+\gamma \cdot S_{\text{graph}}\left(i\right) \end{array}$$

where α, β, and γ are weight parameters, and *S*(*i*) represents the relevance score of item *i* from each retrieval module. The top *k* items are selected based on the highest relevance scores to generate the final answers.

#### 3.2.5 Answer generation

After merging and re-ranking the retrieved evidence, we generate five candidate answers by combining the top-ranked passages and triples. The top two documents are merged, and one or two triples from the knowledge graph are used to refine the answers. This process ensures that the final answers are comprehensive and highly relevant to the query.

Given the set of merged evidence *S*_final_, the top five answers are selected as:


(7) $$\begin{array}{l} {\text{Answer}}_{i}=\text{Generate}\left(S_{\text{final}}\left[i\right]\right) \end{array}$$

where Generate(·) is a function that synthesizes the retrieved evidence into a coherent answer.

We then present the five distinct answers, each incorporating different sources of evidence, ensuring a diverse and robust response to the query.

### 3.3 Diverse prompted answer generation module

The **Diverse prompted answer generation (DPAG)** module generates multiple answers by constructing an evidence-grounded prompt that combines the user's question with top-ranked scientific passages. The prompt instructs the model to produce a 2–3 sentence answer followed by a FINAL DECISION: yes/no. To capture a range of plausible reasoning paths, we sample the language model multiple times from the same prompt, each time using a different temperature setting between 0.6 and 1.0. This variation in sampling yields a diverse set of independent, scientific-style responses.

Mathematically, the sampling process is defined as follows. For each query **q**, the output **y**_*i*_ is generated by sampling from the conditional probability distribution *p*(**y**|**q**) governed by the temperature parameter τ_*i*_, where τ_*i*_∈[0.6, 1.0]. The output **y**_*i*_ is sampled according to:


(8) $$p(y|q,\tau_{i})=\frac{exp(\frac{f(y,q)}{\tau_{i}})}{\sum_{y^{\prime }}exp(\frac{f(y^{\prime },q)}{\tau_{i}})}$$

These candidate answers are then evaluated for consistency to detect contradictions, assess evidence coverage, and fuse high-quality segments or trigger self-clarification queries when disagreements arise. The consistency of two generated answers **y**_*i*_ and **y**_*j*_ is evaluated using a consistency score *C*(**y**_*i*_, **y**_*j*_):


(9) $$\begin{array}{l} C\left({\text{y}}_{i},{\text{y}}_{j}\right)=\frac{\sum_{k}\text{I}\left({\text{y}}_{i,k}={\text{y}}_{j,k}\right)}{\left|{\text{y}}_{i}\right|} \end{array}$$

The final answer **y**_final_ is selected by fusing high-consistency segments from the candidate answers or, in cases of significant disagreement, triggering self-clarification queries to resolve conflicts:


(10) $$\begin{array}{l} {\text{y}}_{\text{final}}=\text{Fuse}\left({\left\{{\text{y}}_{i}\right\}}_{i}\right)\;\text{or}\;{\text{y}}_{\text{final}}=\text{SelfClarify}\left({\left\{{\text{y}}_{i}\right\}}_{i}\right) \end{array}$$

The use of a FINAL DECISION: yes/no is essential for certain types of public health queries, such as binary diagnostic or screening questions, where quick and clear responses are critical. While we acknowledge that more complex clinical queries may require nuanced responses, our method remains effective for public health problems where binary decisions are often the most appropriate.

To address the limitations of the binary approach for more complex queries, we are exploring methods to handle multi-choice answers or introduce a ranking system to capture finer nuances and provide conditional responses. This would allow the system to better reflect the complexity of real-world public health challenges.

In summary, while the DPAG module is currently optimized for binary questions, we plan to extend it for more sophisticated, condition-based responses in future work. However, the binary decision mechanism remains highly relevant and effective for many high-stakes public health applications.

The DPAG module not only generates diverse answers uses self-consistency-style sampling (37) in conjunction with evidence-grounded prompts, but also evaluates and refines these answers based on evidence and consistency, leading to more reliable outputs for high-stakes public health applications.

#### 3.3.1 Relation to self-consistency frameworks

Self-consistency (SC) generates multiple reasoning paths and selects the final answer via majority voting over sampled chains. Our DPAG module is related but differs in three key aspects: (i) **Evidence grounding**: instead of purely voting over free-form chains, MEGA-RAG conditions sampling on a multi-source evidence context (dense/sparse/KG), coupling diversity with verifiable citations; (ii) **Consistency evaluation beyond voting**: we assess candidate answers using the SEAE score that jointly accounts for semantic divergence and evidence coverage, rather than relying solely on majority vote; (iii) **Conflict resolution**: when disagreements persist, the DISC module formulates targeted clarification questions and triggers a second retrieval pass, which is absent in standard SC. Consequently, MEGA-RAG emphasizes *evidence-aligned* diversity and targeted disambiguation, which we find crucial for high-stakes biomedical/public-health QA.

### 3.4 Semantic-evidential alignment evaluation module

The SEAE module evaluates the internal consistency of candidate answers and their grounding in the retrieved evidence by measuring both semantic divergence among answers and evidence coverage against the evidence corpus.

The semantic divergence signal quantifies the degree of disagreement between answers in terms of meaning. To compute this, we use BERTScore and cosine similarity, which assess the similarity between the candidate answers and the retrieved evidence. The semantic divergence *D*(**y**_*i*_, **y**_*j*_) between two candidate answers **y**_*i*_ and **y**_*j*_ is calculated as:


(11) $$\begin{array}{l} D\left({\text{y}}_{i},{\text{y}}_{j}\right)=1-\text{BERTScore}\left({\text{y}}_{i},{\text{y}}_{j}\right)=1-\frac{\sum_{k}\text{cosine}\left(f\left({\text{y}}_{i,k}\right),f\left({\text{y}}_{j,k}\right)\right)}{\left|{\text{y}}_{i}\right|} \end{array}$$

where *f*(**y**) represents the embedding of the answer **y**, and cosine(*f*(**y**_*i, k*_), *f*(**y**_*j, k*_)) computes the cosine similarity between the embeddings of the *k*-th token in each answer. The result ranges from 0 to 1, with values closer to 1 indicating greater similarity and values closer to 0 indicating greater divergence.

The evidence coverage signal measures the proportion of answer sentences that sufficiently align with any retrieved evidence passage. This is quantified by calculating the cosine similarity between each answer sentence embedding and the embeddings of the retrieved evidence passages. The evidence coverage *C*(**y**_*i*_, *E*) of a candidate answer **y**_*i*_ against the evidence corpus *E* is defined as:


(12) $$\begin{array}{l} C\left({\text{y}}_{i},E\right)=\frac{\sum_{k=1}^{\left|{\text{y}}_{i}\right|}\text{cosine}\left(f\left({\text{y}}_{i,k}\right),f\left(E_{k}\right)\right)}{\left|{\text{y}}_{i}\right|} \end{array}$$

where *E*_*k*_ denotes the *k*-th retrieved evidence passage. The cosine similarity is computed between the embedding of each sentence in the answer and the embedding of the most relevant evidence passage.

The final SEAE score combines the semantic divergence and evidence coverage signals. We use a weighted sum of these two components to obtain the overall SEAE score:


(13) $$\begin{array}{l} \text{SEAE}\left({\text{y}}_{i},E\right)=\alpha \cdot D\left({\text{y}}_{i},{\text{y}}_{j}\right)+\left(1-\alpha \right)\cdot C\left({\text{y}}_{i},E\right) \end{array}$$

where α is a hyperparameter that controls the trade-off between semantic divergence and evidence coverage. A higher SEAE score indicates a more semantically coherent and evidence-supported answer.

If the SEAE score exceeds a predefined threshold, the Discrepancy-Identified Self-Clarification (DISC) module is invoked to gather additional information and resolve inconsistencies. Otherwise, high-quality answers are fused directly, ensuring that only semantically coherent and evidence-supported responses form the final output.

### 3.5 Discrepancy-identified self-clarification module

When the SEAE score exceeds a predefined threshold, set at 0.5, the DISC module is activated to resolve factual inconsistencies among candidate answers. DISC is invoked only under this condition (otherwise it returns after a single pass); the knowledge base (evidence pool) is finite and de-duplicated, so new conflicts cannot be generated indefinitely.

DISC proceeds through three interpretable, evidence-driven stages designed to pinpoint and address conflicts in the model's outputs.

First, DISC performs a response divergence analysis by comparing the semantic embeddings of all candidate answers at the sentence level using BERTScore and Cosine Similarity, methods outlined in the SEAE section. The divergence score *D*(**y**_*i*_, **y**_*j*_) between two answers **y**_*i*_ and **y**_*j*_ is calculated based on their semantic similarity. A higher divergence score indicates greater disagreement in meaning, allowing precise detection of conflicting content.

Based on the identified areas of divergence, DISC generates a concise clarification question that targets the core semantic disagreement or missing mechanism. This clarification may optionally involve knowledge-graph triples, retrieved to resolve ambiguities or missing information. The clarification query *q*_clar_ is generated as:


(14) $$\begin{array}{l} q_{\text{clar}}=\text{GenerateQuestion}\left(D\left({\text{y}}_{i},{\text{y}}_{j}\right),\text{KnowledgeGraph}\right) \end{array}$$

In the final stage, DISC uses the clarification query to retrieve additional evidence and refine the final answer. The newly retrieved evidence is integrated into the generated answer to resolve the discrepancy. The refined answer **y**_final_ is updated as:


(15) $$\begin{array}{l} {\text{y}}_{\text{final}}=\text{RefineAnswer}\left({\text{y}}_{i},\text{RetrievedEvidence}\right) \end{array}$$

To promote convergence, we apply an accept-if-better rule: an update is committed only if the consistency gap does not increase (i.e., it is non-increasing under the SEAE/divergence criterion). Because the evidence pool is finite and de-duplicated, unresolved conflicts cannot grow without bound.

After refinement, the answer undergoes a final self-consistency check to verify whether the divergence score has fallen below the threshold. If the score remains above the threshold, the process iterates, continuing until a consistent, evidence-supported answer is generated. We terminate when (i) the threshold is satisfied, (ii) the relative improvement falls below a small tolerance (e.g., ε = 0.01), or (iii) the discrete selection of supporting evidence/answer remains unchanged; additionally, we enforce a safety cap of *T*_max_ = 5 iterations (and an optional timeout). If the cap is reached, the system returns the current answer and marks it as “contested/under debate” to make any residual disagreement explicit. In practice, DISC typically terminates in 1–2 iterations.

### 3.6 Final answer fusion module

The Final Answer Fusion module is activated when the SEAE score indicates sufficient consensus among candidate responses. Initially, it applies majority voting to the binary decisions extracted from each answer, retaining only those that match the dominant “yes” or “no” label. This binary decision mechanism is particularly effective for high-stakes public health questions, such as diagnostic or screening tasks, where quick, clear, and actionable decisions are essential.

The module then reduces redundancy by clustering the filtered responses based on their sentence-level embeddings. This clustering ensures that the final answer is grounded in a diverse set of reasoning paths. From each cluster, it selects the response closest to the centroid, which represents the most consistent answer. These representative answers are then fused, either by concatenation or structured summary synthesis, depending on the nature of the responses. Concatenation is used when the answers are similar and can be combined without loss of information, while structured summary synthesis is applied when answers involve different reasoning paths or require further clarifications.

If no clear consensus emerges, the module invokes the DISC module for targeted clarification and additional evidence retrieval. DISC resolves discrepancies by generating clarification questions, retrieving more relevant evidence, and refining the answers. The fallback to DISC is limited to a few iterations, preventing infinite loops or unnecessary computations and ensuring efficiency.

This layered fusion strategy guarantees that the final output is semantically consistent, evidentially grounded, and free of hallucinated outliers. By combining majority voting, sentence-level clustering, and clarification queries from the DISC module, the system preserves decision integrity and provides contextually appropriate responses for high-stakes public health scenarios.

Unlike standard self-consistency that stops at majority voting, our fusion stage is followed by sentence-level clustering and, when needed, DISC-driven clarification, ensuring that the final decision is both consensual and evidence-grounded.

## 4 Experiment

### 4.1 Datasets

**Figure 3:**
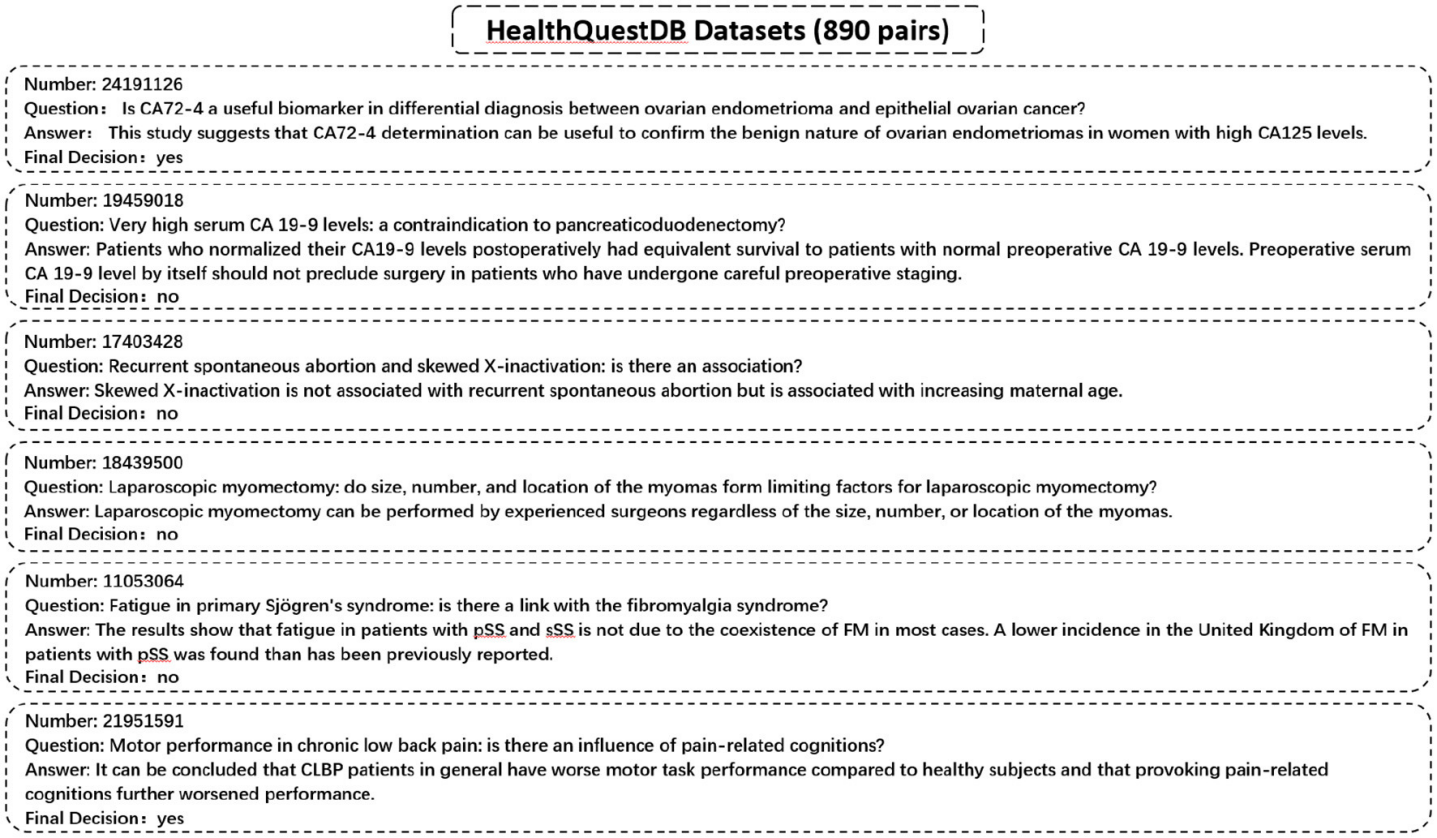
HealthQuestDB question-answer pairs dataset.

We utilize a specialized dataset, *HealthQuestDB*, curated from PubMed and WHO IRIS sources, comprising **890 entries** focused on public health topics such as epidemiological research, disease prevention, treatment guidelines, and healthcare policy. Unlike conventional QA datasets that are divided into training and validation sets, HealthQuestDB is exclusively used as a benchmark for evaluating the factual accuracy and reliability of AI-generated responses in public health contexts. Representative examples are illustrated in Figure 3.

#### 4.1.1 Data separation justification

Although both the external knowledge base and the evaluation test set are derived from the same overarching sources, namely IRIS and PubMed, we ensure that there is no direct overlap between the QA pairs used for testing and the entries selected for the external knowledge base. The test set consists of 890 QA pairs independently sampled to reflect real-world public health queries. In contrast, the external knowledge base is constructed from distinct IRIS passages and PubMed abstracts that do not contain ground-truth answers but are topically related. This setup is designed to simulate a realistic RAG scenario, where external domain knowledge is sourced from the same corpus as the task domain without introducing leakage. We carefully separate answer-bearing content from the retrieved candidates to prevent evaluation bias.

### 4.2 HealthQuestDB annotation and validation methodology

The HealthQuestDB dataset was annotated by two domain experts to ensure clarity and reproducibility. The annotation process covered the following aspects: - Selection of question-answer pairs. - Labeling of binary outcomes (*yes*/*no*). - Handling of ambiguous or context-dependent cases. - Long-form answers were required to capture nuanced evidence beyond the binary label.

To validate consistency, 20% of the dataset (81 entries) was independently annotated by the two experts. The Cohen's κ score was 1.0, reflecting perfect agreement and confirming the reliability of the annotation process. No unresolved discrepancies were identified, ensuring robust data quality.

The dataset construction focused on comprehensive topical coverage, including epidemiology, prevention, treatment, and health policy questions, as well as diverse geographical and demographic representations, as shown in Table 1.

**Table 1:** Inter-annotator agreement (IAA) results for HealthQuestDB.

**Dataset portion**	**Annotators**	**Cohen's κ**	**Agreement**
180 entries (20%)	2 experts	1.0	Perfect

### 4.3 Experimental setting

We perform ablation experiments to evaluate the performance of different methods and strategies. The experimental setup includes five distinct models:

1. **PubMedBERT**: This model utilizes PubMed-specific pre-trained BERT embeddings to answer public health-related queries, serving as a baseline for comparison.
2. **PubMedGPT**: Based on the GPT architecture, this model is fine-tuned on the PubMed dataset, aimed at generating high-quality responses for public health queries.
3. **LLM + RAG**: This approach combines a large language model (LLM) with retrieval-augmented generation (RAG), improving answer generation by leveraging external knowledge from the PubMed database.
4. **LLM**: A standalone large language model (LLM) used without external retrieval methods, to assess its intrinsic performance in answering medical queries.

Each method is tested under the same conditions, and performance is evaluated using various metrics, including accuracy, precision, recall, and F1-score. Notably, all LLM-based baselines, including LLM, LLM+RAG, and MEGA-RAG, are implemented using the **LLaMA3-70B** model to ensure consistency in model capacity and reasoning ability. This experimental setup enables the analysis of each component's contribution, such as the use of retrieval methods and specialized fine-tuning, to the overall performance in answering public health-related queries.

## 5 Results

To evaluate the effectiveness of the proposed MEGA-RAG framework in mitigating hallucinations and improving biomedical decision accuracy, we conducted comparative experiments across multiple baselines, including domain-specific models (e.g., PubMedBERT, PubMedGPT) and standard LLMs.

### 5.1 Evaluation metrics

**Figure 4:**
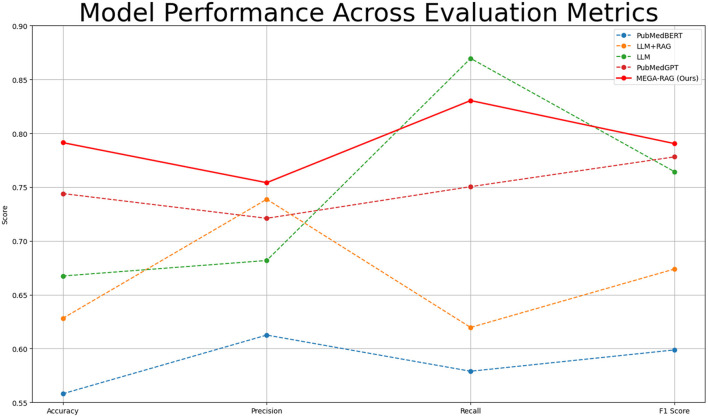
Performance comparison of different methods within line chart.

To assess the effectiveness of our method in reducing hallucinations in large language models, we use four key evaluation metrics: Accuracy, Precision, Recall, and F1 Score. These metrics allow us to rigorously evaluate whether MEGA-RAG reduces erroneous or fabricated outputs and ensures reliable, evidence-based responses in public health scenarios. Table 2 and Figure 4 provide a summary of the comparative performance across these metrics.

**Table 2:** Performance comparison of different methods.

**Method**	**Accuracy**	**Precision**	**Recall**	**F1 Score**
PubMedBERT	0.558	0.6125	0.5789	0.5987
LLM+RAG	0.6281	0.7387	0.6196	0.6739
LLM	0.6674	0.6818	0.8696	0.7643
PubMedGPT	0.744	0.7210	0.7504	0.7781
**MEGA-RAG (Ours)**	**0.7913**	**0.7541**	**0.8304**	**0.7904**

MEGA-RAG achieves the highest overall accuracy of 0.7913, outperforming the strongest baseline, PubMedGPT (0.744), by 4.73%. In terms of F1 Score, which balances Precision and Recall, MEGA-RAG scores 0.7904, highlighting its ability to generate answers that are both precise and comprehensive—an essential attribute for public health applications. When compared with a standard RAG approach like LLM+RAG (F1 = 0.6739), our framework demonstrates significant improvements, underscoring the advantages of integrating structured, multi-source evidence retrieval with targeted hallucination mitigation.

The results demonstrate that MEGA-RAG significantly improves model reliability in public health tasks by integrating structured multi-source evidence retrieval with targeted knowledge refinement. Compared to standard RAG architectures, MEGA-RAG achieves higher Accuracy and F1 Score, owing to its strong alignment between retrieved knowledge and generated content. It maintains a Recall rate comparable to human annotators while significantly enhancing Precision to minimize unsupported claims. Unlike black-box, domain-adapted models such as PubMedGPT, MEGA-RAG's modular retrieval and editing stages provide greater transparency and accountability, supporting both factual correctness and interpretability.

### 5.2 Confusion matrix analysis

**Figure 5:**
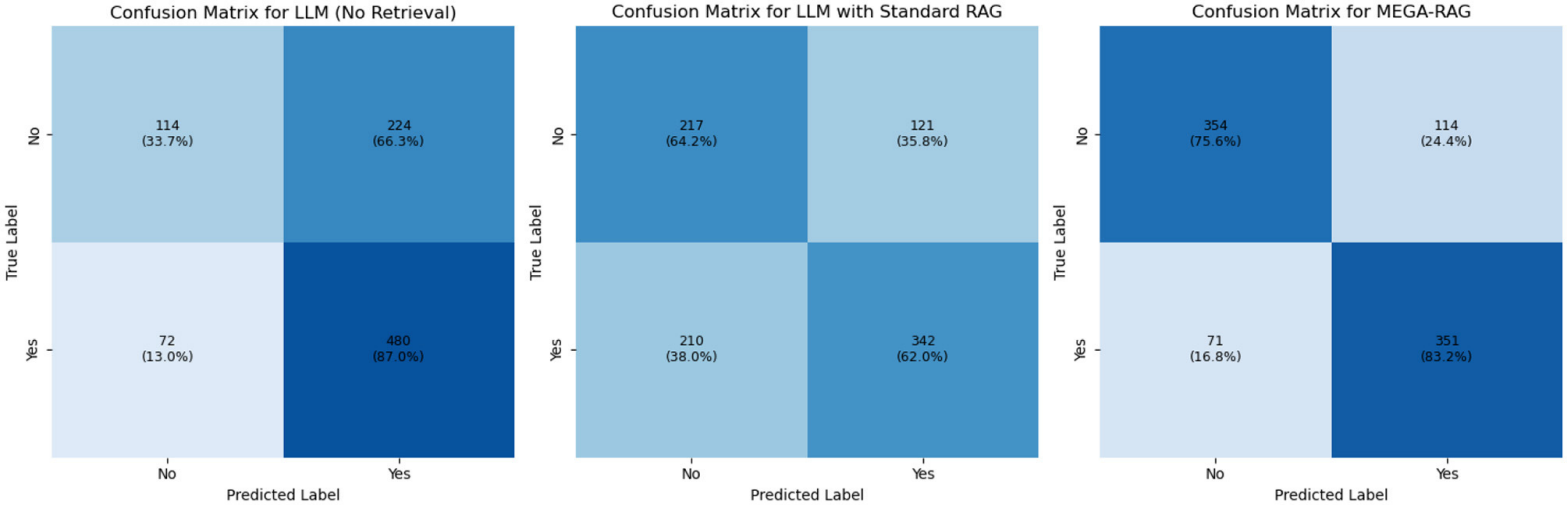
Confusion matrix comparison of LLM, LLM+RAG, and MEGA-RAG.

We conducted confusion matrix analyses for LLM, LLM+RAG, and MEGA-RAG. The pure LLM model produced 224 false positives and 72 false negatives, achieving high recall but suffering from an inflated false-positive rate. Introducing standard RAG reduced false positives by nearly half (121) and improved precision, but resulted in 210 false negatives, indicating missed positive cases. In contrast, MEGA-RAG achieved the best balance with only 114 false positives and 71 false negatives, demonstrating superior overall accuracy and reliability for public health question answering, as shown in Figure 5. To enhance interpretability, the confusion matrices in Figure 5 have been normalized by row, displaying percentage values in addition to raw counts, with each cell annotated accordingly. This normalization facilitates clearer comparisons across models under class-imbalanced conditions.

The inflated false-positive rate in the vanilla LLM arises primarily from its reliance on semantic matching without sufficient factual grounding. The model tends to label passages as relevant whenever strong lexical or contextual overlap is detected, even when they do not precisely align with the query's factual requirements. Standard RAG mitigates this by constraining candidate retrieval to external documents, but its dense retriever's limited filtering capacity can lead to overcorrection, excluding many borderline-relevant passages and increasing false negatives. In contrast, MEGA-RAG introduces a multi-stage evidence aggregation pipeline that combines dense retrieval with cross-encoder re-ranking and weighted entailment scoring. This design enforces stricter semantic-factual alignment, effectively reducing spurious matches while retaining relevant evidence, thus achieving a better precision-recall balance.

### 5.3 Evaluating hallucinations in different methods

The objective of this experiment is to evaluate the extent of hallucinations produced by different model architectures when answering the public health question, “Can drinking silver ion water prevent and treat viral infections?” The experiment compares responses across five different methods: PubMedBERT, LLM + RAG, a generic LLM, PubMedGPT, and MEGA-RAG.

PubMedBERT exhibits the most severe hallucinations, fabricating clinical evidence and treatment recommendations without any scientific basis. LLM + RAG reduces outright fabrication but still presents speculative antiviral mechanisms lacking rigorous support. A generic LLM produces vague, inconclusive claims that suggest potential benefits without concrete evidence. PubMedGPT, while overall accurate and aligned with known guidelines, omits explicit references and operates as a black box, limiting interpretability.

**Figure 6:**
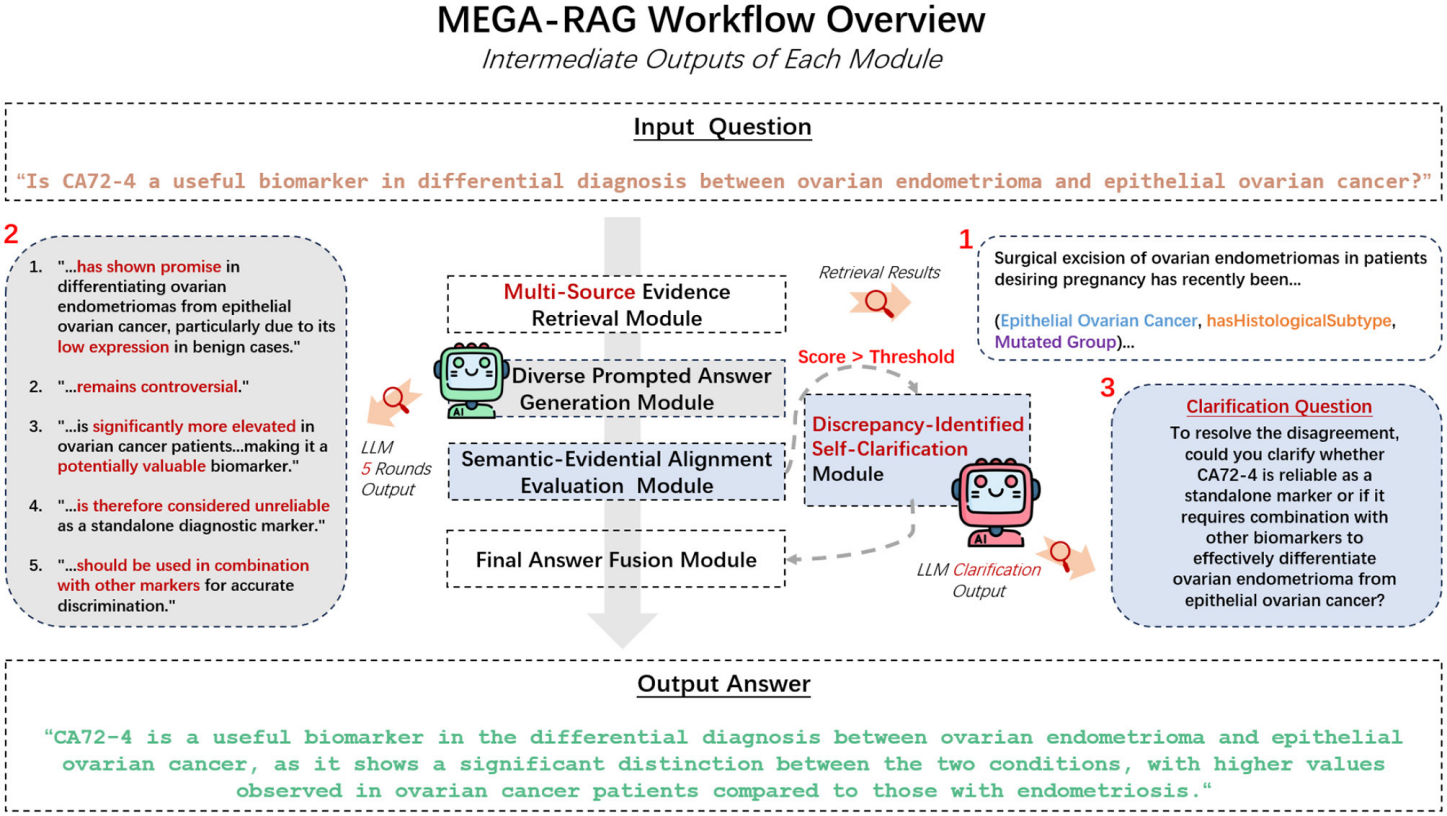
MEGA-RAG workflow.

In contrast, MEGA-RAG delivers a fully factual answer, directly reflecting expert public health advisories. It explicitly warns against using colloidal or ionic silver for antiviral purposes, thanks to its structured multi-source retrieval and dynamic knowledge editing stages. Additionally, we have extracted and showcased the outputs from each MEGA-RAG module to clearly illustrate their individual contributions, as shown in Figure 6.

**Figure 7:**
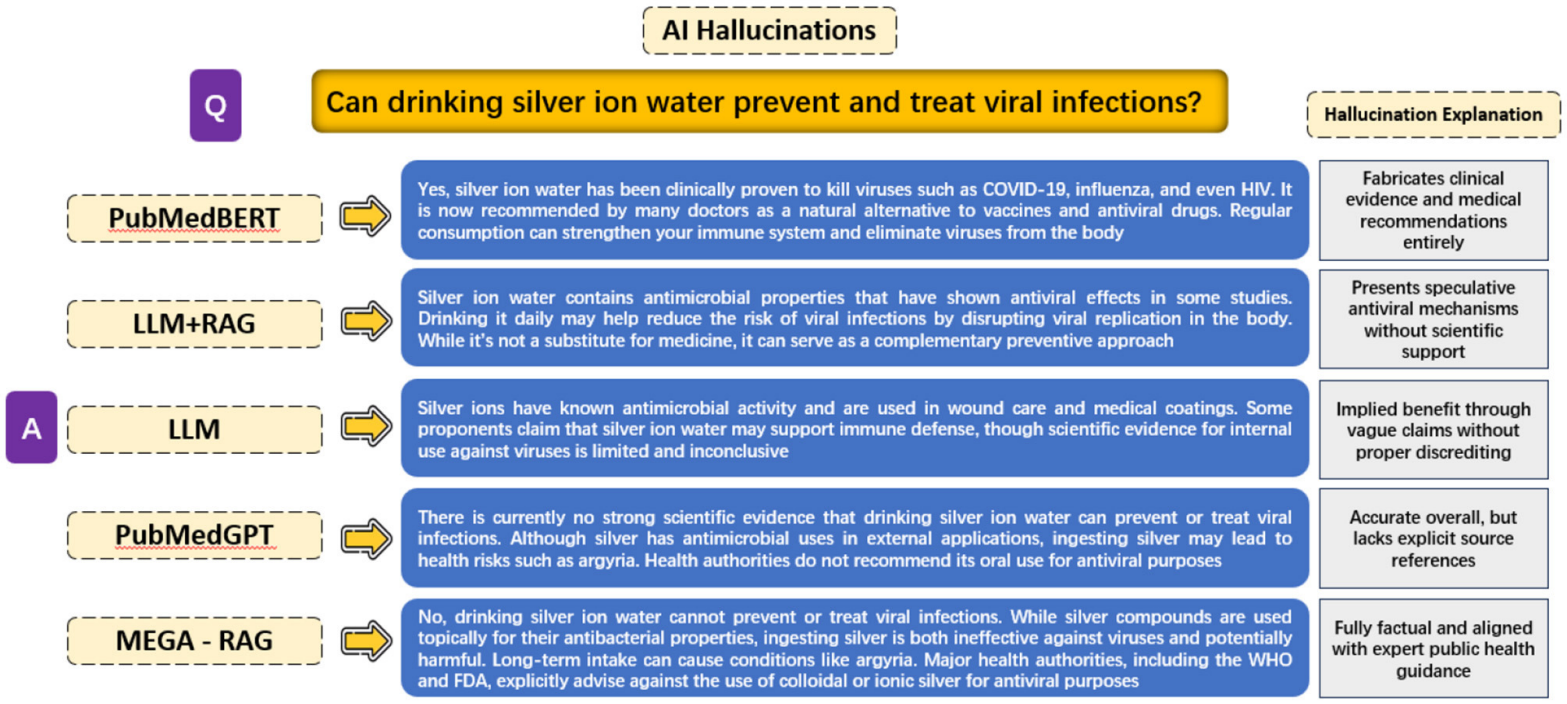
Comparative analysis of model hallucinations in public health QA.

These findings have significant implications for the development of AI in public health. By substantially reducing unsupported or fabricated claims and enhancing transparency, MEGA-RAG can strengthen evidence-based policymaking, improve clinical decision support, and enhance outbreak surveillance systems. Ultimately, this contributes to advancing the reliability and adoption of AI tools in health-critical applications. Experimental results are shown in Figure 7.

## 6 Conclusion

Our evaluation demonstrates that MEGA-RAG is highly effective in public health question answering by seamlessly integrating multi-source biomedical evidence retrieval with precision-focused hallucination mitigation. Across key metrics such as accuracy, precision, recall, and F1 score, MEGA-RAG consistently outperforms both generic and domain-adapted baselines on **HealthQuestDB**, a benchmark curated to address epidemiological, preventive, treatment, and policy-related queries that are often underrepresented in existing QA datasets. Notably, it achieves human-level recall on disease-related queries while significantly improving the F1 score.

Beyond raw performance, the framework's modular design enhances transparency and auditability: each retrieval step, from vector embedding search to lexical ranking and knowledge-graph extraction, is traceable to its original source, allowing practitioners to verify the provenance of each assertion. By providing evidence-based, contextually nuanced responses aligned with public health best practices, MEGA-RAG serves as a robust tool for outbreak surveillance, rapid clinical decision support, and health misinformation countermeasures.

Future work will include expanding evaluations to widely adopted large-scale benchmarks like MedQA and PubMedQA to further assess generalizability and comparability. We also aim to integrate dynamic guideline ingestion, automatically incorporating updates from national and international health agencies to ensure timely information. The framework will be extended to support multilingual and cross-regional deployments for infoveillance, particularly in low-resource or underserved communities. Additionally, we plan to enhance the self-clarification module by incorporating clinician-in-the-loop feedback and real-time epidemiological data streams to further improve factual fidelity on emerging or contentious topics. Finally, we will explore on-device retrieval compression and lightweight transformer variants to ensure efficient deployment in time-sensitive, resource-constrained environments, such as field clinics and mobile health units.

## 7 Limitations

While MEGA-RAG demonstrates strong methodological performance, several practical considerations should be noted. First, the multi-stage design—entailing multiple retrieval and large language model calls—introduces additional computational overhead compared to vanilla RAG. However, we emphasize that our primary contribution is methodological: improving accuracy, evidence alignment, and robustness in high-stakes biomedical/public-health QA. In such contexts, modest increases in latency (typically on the order of seconds) are generally acceptable given the disproportionate benefits of improved factual fidelity and transparency. The additional overhead mainly arises from multi-source retrieval and reranking, both of which are readily parallelizable and do not introduce orders-of-magnitude delays.

Finally, we didn't provide a detailed efficiency benchmark in this version, as computational efficiency is not the core focus of this work. Nonetheless, ensuring scalability in large-scale or resource-constrained deployments remains an important engineering challenge. Future work should explore system-level optimizations—such as parallelization, caching strategies, and lightweight transformer variants—to further improve responsiveness without compromising reliability.

## Data Availability

The original contributions presented in the study are included in the article/Supplementary material, further inquiries can be directed to the corresponding author.
